# An Integrated, Scalable, Electronic Video Consent Process to Power Precision Health Research: Large, Population-Based, Cohort Implementation and Scalability Study

**DOI:** 10.2196/31121

**Published:** 2021-12-08

**Authors:** Clara Lajonchere, Arash Naeim, Sarah Dry, Neil Wenger, David Elashoff, Sitaram Vangala, Antonia Petruse, Maryam Ariannejad, Clara Magyar, Liliana Johansen, Gabriela Werre, Maxwell Kroloff, Daniel Geschwind

**Affiliations:** 1 Institute for Precision Health David Geffen School of Medicine at UCLA Los Angeles, CA United States; 2 Center for SMART Health Institute for Precision Health David Geffen School of Medicine at UCLA Los Angeles, CA United States; 3 Department of Pathology and Laboratory Medicine David Geffen School of Medicine at UCLA Los Angeles, CA United States; 4 Department of Medicine David Geffen School of Medicine at UCLA Los Angeles, CA United States; 5 Embedded Clinical Research and Innovation Unit Clinical and Translational Science Institute David Geffen School of Medicine at UCLA Los Angeles, CA United States

**Keywords:** biobanking, precision medicine, electronic consent, privacy, consent, patient privacy, clinical data, eHealth, recruitment, population health, data collection, research methods, video, research, validation, scalability

## Abstract

**Background:**

Obtaining explicit consent from patients to use their remnant biological samples and deidentified clinical data for research is essential for advancing precision medicine.

**Objective:**

We aimed to describe the operational implementation and scalability of an electronic universal consent process that was used to power an institutional precision health biobank across a large academic health system.

**Methods:**

The University of California, Los Angeles, implemented the use of innovative electronic consent videos as the primary recruitment tool for precision health research. The consent videos targeted patients aged ≥18 years across ambulatory clinical laboratories, perioperative settings, and hospital settings. Each of these major areas had slightly different workflows and patient populations. Sociodemographic information, comorbidity data, health utilization data (ambulatory visits, emergency room visits, and hospital admissions), and consent decision data were collected.

**Results:**

The consenting approach proved scalable across 22 clinical sites (hospital and ambulatory settings). Over 40,000 participants completed the consent process at a rate of 800 to 1000 patients per week over a 2-year time period. Participants were representative of the adult University of California, Los Angeles, Health population. The opt-in rates in the perioperative (16,500/22,519, 73.3%) and ambulatory clinics (2308/3390, 68.1%) were higher than those in clinical laboratories (7506/14,235, 52.7%; *P*<.001). Patients with higher medical acuity were more likely to opt in. The multivariate analyses showed that African American (odds ratio [OR] 0.53, 95% CI 0.49-0.58; *P*<.001), Asian (OR 0.72, 95% CI 0.68-0.77; *P*<.001), and multiple-race populations (OR 0.73, 95% CI 0.69-0.77; *P*<.001) were less likely to participate than White individuals.

**Conclusions:**

This is one of the few large-scale, electronic video–based consent implementation programs that reports a 65.5% (26,314/40,144) average overall opt-in rate across a large academic health system. This rate is higher than those previously reported for email (3.6%) and electronic biobank (50%) informed consent rates. This study demonstrates a scalable recruitment approach for population health research.

## Introduction

Informed consent for the research use of data and biological specimens is an essential and critical component of a robust program in precision medicine [1-3]. Although the common rule [4] considers the research use of deidentified tissue as “not human subjects” research, the National Institutes of Health (NIH) Genomic Data Sharing Policy expects consent to be obtained for future research use and broad data sharing, even if biospecimens are deidentified [5,6].

The 2017 revision to the common rule includes a new category of regulatory broad consent that provides more flexibility for researchers to consent participants for the storage, biobanking, and secondary research use of identifiable information or biospecimens [4]. Further, many advocates and ethicists have articulated an obligation to communicate that remnant tissue may be used for research and that researchers should proactively obtain informed consent [7,8]. Patients have also expressed a desire to have their preferences dictate the use of clinical specimens for research [8,9]. From this perspective, large-scale precision medicine programs that hope to engender greater trust and foster external collaborations, including collaborations with commercial entities and federal agencies, should consider proactively structuring their consent processes to include these key aspects of a broader and more informative consent process.

The emergence of digital health has also played a significant role in defining precision health approaches to obtaining electronic consent [10-12]. Obtaining in-person paper consent is often resource intensive, is not easily scalable, and precludes digital responses from being incorporated in the electronic health record (EHR) and laboratory information management systems [10-14]. Given that precision medicine research requires large-scale patient engagement, innovations in consent processes and public education [1,15] are required. Animated video consent approaches have been effective in providing comprehensive information and improving participants’ understanding of content [16,17] and have thus provided an opportunity to more effectively increase the participation of traditionally underrepresented communities, as content can be tailored to participant groups.

The Engaging University of California Stakeholders for Biorepository Research (EngageUC) Consent Trial [18] was conducted to harmonize biobanking policies and procedures across 5 University of California medical campuses with the help of NIH Clinical and Translational Science Institutes (CTSI). The investigators developed an evidence-based approach that allowed for iterative stakeholder engagement to develop efficient biobank operations and equitable governance processes. The key themes that emerged were that the public should be educated about biobanking; the source of consent content should be considered knowledgeable and trustworthy; the process should be low stress and provide an opportunity to obtain answers to questions; the format and language should be easy to understand; and stakeholders, including the community, should play a role in informing and advising the institution. This framework provides a pathway for addressing the technical and ethical challenges that must be resolved to ensure that biorepositories continue to support translational research in ways that are inclusive of the populations they serve.

In 2016, the University of California, Los Angeles (UCLA), Institute for Precision Health (IPH) launched their ATLAS Community Health Initiative to engage a diverse sample of patients across UCLA Health in precision health research. The goal was to create a powerful and robust clinical and genomic data resource for cutting-edge translational research. This required innovative and scalable solutions for sustaining the program’s rapid growth. The IPH partnered with this study’s team to further develop and pilot their electronic universal video consent (UCON) process for biological samples, which was used to power the ATLAS precision health biobank.

In the development phase of this program, we leveraged the learnings from the EngageUC Consent Trial [18] and engaged community members across the greater Los Angeles region and internal stakeholders across the UCLA Health system, David Geffen School of Medicine at UCLA, the IPH, and CTSI as part of the initial development and pilot of the UCON process in targeted clinics at UCLA Health [19]. This “one-time” consent process gives all adult patients the autonomy to choose whether they want their deidentified biospecimens and clinical data to be made available for research.

In this paper, we describe phase 2 of the ATLAS Community Health Initiative, which focuses on the operational implementation and scalability of the UCON process. This includes its interoperability with the EHR and laboratory information management systems that power the UCLA ATLAS precision health biobank. We expanded the animated UCON process to 18 UCLA Health clinics across the Los Angeles region to test its scalability as an enterprise solution.

## Methods

### Study Setting

UCLA Health is one of the most diverse, comprehensive, and leading academic medical centers in Southern California. Ranked first in California and third in the nation, the UCLA Health system is comprised of a number of hospitals, including Ronald Reagan UCLA Medical Center and UCLA Santa Monica Medical Center, and an extensive primary care network of >180 medical offices in the greater Los Angeles area. This study was approved by the UCLA Institutional Review Board (institutional review board number: 15-001395IRB) with a waiver of written informed consent.

### Electronic Video Consent

**Figure 1:**
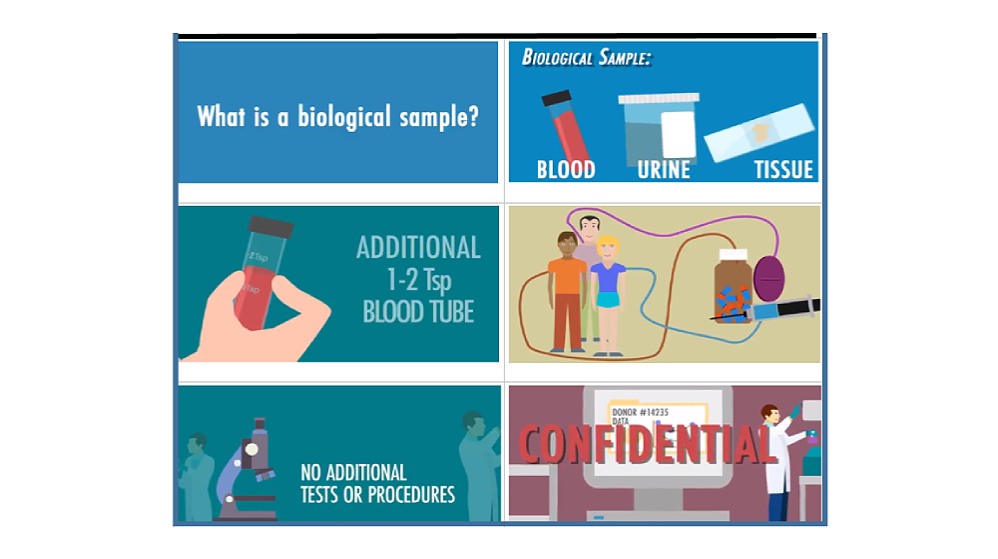
Representative screenshots of universal consent animated videos. Tsp: teaspoon.

The electronic consent videos were designed to be self-administered, be fast (around 7 minutes), and meet the NIH consent threshold [19]. These fully animated (cartoon-like) videos were developed to better communicate content to lay audiences (Figure 1). The videos [20] were available in English and Spanish and included voice-overs. All of the essential components of regulatory broad informed consent were included in the videos. Our study team assembled a community advisory board (CAB) consisting of 11 respected leaders who were highly involved with organizations in the Los Angeles region that understood our diverse communities and represented their perspectives. The members were racially diverse (African American: n=2; Latinx: n=2; Asian American: n=1; Native American: n=1; Persian American: n=1; White and Non-Hispanic: n=4) and equitable with respect to gender (male: n=5; female: n=6). The CAB reviewed the results of a convenience sample of 117 patients who underwent cognitive testing. Operational feasibility was also tested with 625 additional patients. The CAB made the recommendation to move forward with the expansion to the broader UCLA Health population [19].

### Consent Choices

With regard to patients’ opt-in and opt-out statuses, patients could choose to (1) opt in to share their remnant samples plus a dedicated blood sample, (2) opt in to share remnant tissues only, or (3) opt out altogether. We also tracked the number of patients who opted in and indicated a willingness to be recontacted for future research.

### Workflows

**Figure 2:**
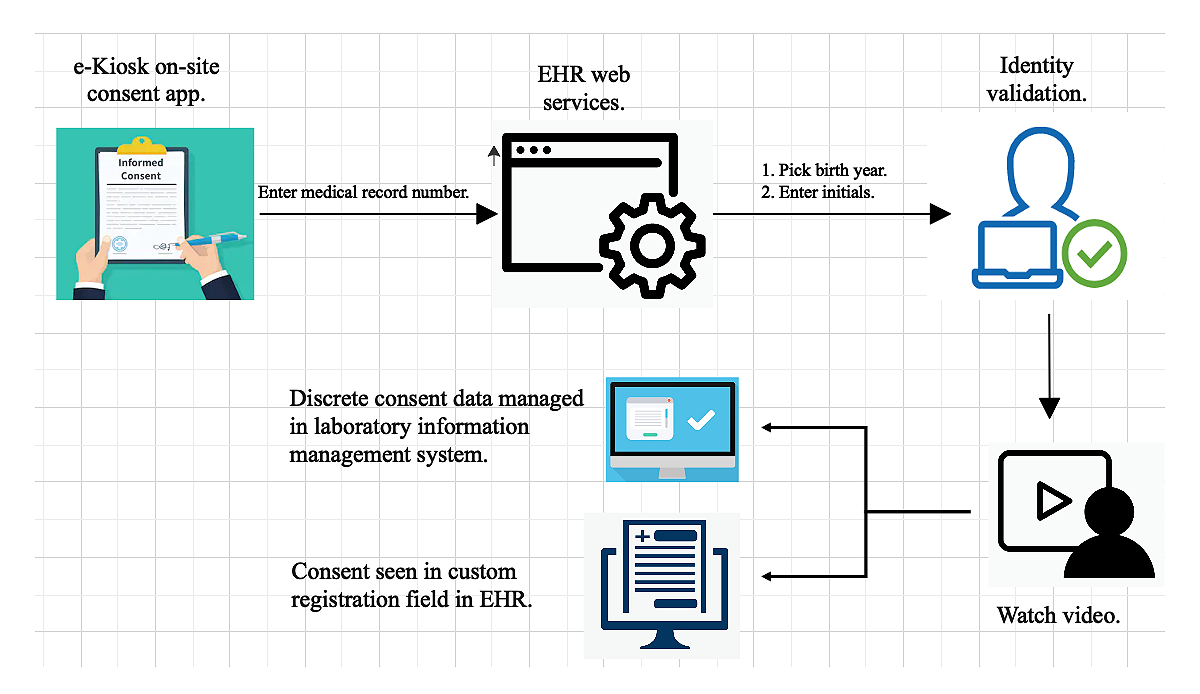
Universal consent workflow. EHR: electronic health record.

The UCON process was deployed in perioperative suites, clinical labs, and ambulatory clinics (Figure 2). In perioperative suites, patients were handed an iPad (Apple Inc), were instructed to watch the UCON video, and provided their consent decision. The iPad was then collected by nurses in the perioperative area. If a patient consented to providing an extra blood tube, the nurses placed the order in the EHR per the protocol. Patients typically waited for 30 to 60 minutes prior to their procedure, which provided ample time for completing the consent process. All extra tubes were automatically routed to the precision health biobank for DNA extraction and storage. In clinical labs, patients were handed an iPad by the front desk staff. Patients typically waited for 10 to 15 minutes prior to a lab draw and were able to watch the 5-minute UCON video and complete the consent process. Lastly, patients who were consented in ambulatory clinics completed the consent process at a self-service kiosk or by using an iPad before, during, or after a clinic visit. Patients waited for 10 to 15 minutes before moving to an exam room and another 10 to 15 minutes prior to meeting with a care provider, which provided ample time for completing the consent process. Once remnant (leftover) tissue samples became available for patients who opted in, these samples were accessioned into the ATLAS precision health biobank. DNA was extracted from whole blood and genotyped. Afterward, the remaining DNA was stored in the biobank.

### Documentation and Tracking Within the EHR

After consent completion, consent decisions were transmitted to the precision health biobank’s laboratory information management system (Biomaterial Tracking Management System; Daedalus Inc). PDF files containing patients’ UCON completion statuses were sent to the EHR (Epic Systems Corporation) [21], which generated a receipt in real time in a custom registration field. The receipt ensured that patients would not be asked to complete the consent process a second time. Clinical staff used this field to determine which patients were eligible to complete the process.

### Data Collection

The following data were collected from all patients who underwent the UCON process: sociodemographic information; comorbidity statuses based on the Charlson Comorbidity Index [22]; health utilization data, including ambulatory visits, emergency room visits, and hospital admissions; and consent decisions. With the exception of consent decisions, the data were all collected from the EHR by an honest broker who merged and deidentified the data via an institutional review board–approved process.

### Statistical Analysis

Descriptive statistics were computed for all study variables. Quantitative variables were summarized by using means, SDs, and quartiles, while categorical variables were summarized by using frequencies and percentages. Sample characteristics were reported for the cohort that completed the consent process and for the larger UCLA Health patient population to determine whether there was sampling bias within and across clinics. US census data were used to obtain comparable summaries (where available) for residents of Los Angeles County.

Comparisons were made among the three workflows. Quantitative variables were compared by using 1-way analysis of variance *F* tests, and categorical variables were compared by using chi-square tests or Fisher exact tests, as appropriate. Consent status, which was categorized as *consented* or *declined*, was modeled via logistic regression.

A stepwise variable selection procedure was used to select a subset of predictor variables for inclusion in the model. This involved using entry and exit criteria for variables with a *P* value of <.001. The results of separate models that included 1 predictor at a time, as well as those of a multivariate model that combined all selected predictors, were reported. Odds ratios (ORs), 95% CIs, and *P* values were used to summarize model results. Model fit was evaluated by using the area under the curve. Statistical significance was defined as a *P* value of <.05. Cohort summaries and comparisons among cohorts were performed by using R version 3.5.0 (R Foundation for Statistical Computing). Logistic regression modeling was performed by using SAS version 9.4 (SAS Institute).

## Results

### Demographic Comparisons

The IPH consented 40,144 participants from March 2017 through June 2019 (Table 1). Of these participants, 26,314 (65.5%) opted into the ATLAS precision health biobank program. The demographics were representative of the UCLA Health population. ATLAS patients were only slightly older than the general UCLA Health population and much older than the Los Angeles County population; 30.2% (12,123/40,144) of ATLAS participants were aged ≥65 years, whereas 27.5% (160,479/583,282) and 16.6% of the UCLA Health and Los Angeles County populations, respectively, were aged ≥65 years. The percentages of African Americans (2558/40,144, 6.4%) and Hispanic individuals (6055/40,144, 15.1%) in the ATLAS cohort were higher than those in the UCLA Health population (African Americans: 27,338/583,282, 4.7%; Hispanic individuals: 64,234/583,282, 11%) but lower than those in the Los Angeles County population (9.1% and 48.5%, respectively). The Los Angeles County data reflect census data from 2019, which only provides rates [23].

**Table 1:** Demographic and clinical characteristics of the cohort and reference populations.

Characteristics			Patients who completed the consent process (N=40,144)		UCLA^a^ Health population (N=583,282)		Los Angeles County population^b^
Age (years), mean (SD)			53.1 (17.5)		51.6 (19.1)		—^c^
Aged ≥65 years, n (%)			12,123 (30.2)		160,479 (27.5)		16.6
Female, n (%)			22,302 (55.6)		333,998 (57.3)		50.7
**Ethnicity, n (%)**							
	Not Hispanic or Latino	32,413 (80.7)		429,327 (73.6)		51.5	
	Hispanic or Latino	6055 (15.1)		64,234 (11)		48.5	
	Other, unknown, or refused	1676 (4.2)		89,721 (15.4)		0	
**Race, n (%)**							
	White	24,840 (61.9)		330,572 (56.7)		71	
	Asian	4667 (11.6)		54,043 (9.3)		15.1	
	Black or African American	2558 (6.4)		27,338 (4.7)		9.1	
	Native Hawaiian or other Pacific Islander	119 (0.3)		1204 (0.2)		0.4	
	American Indian or Alaska Native	115 (0.3)		2282 (0.4)		1.5	
	Multiple races, other, unknown, or refused	7845 (19.5)		167,843 (28.8)		3	
**Marital status, n (%)**							
	Married, significant other, or partner	22,897 (57)		289,364 (49.6)		—	
	Single	12,227 (30.5)		209,016 (35.8)		—	
	Divorced, separated, dissolved, or widowed	4573 (11.4)		52,201 (8.9)		—	
	Other or unknown	447 (1.1)		32,701 (5.6)		—	
**Neighborhood education level (percentage of high school graduates), n (%)**							
	<50%	17,074 (42.5)		209,697 (36)		—	
	>50%	17,785 (44.3)		256,249 (43.9)		—	
	Unknown	5285 (13.2)		117,336 (20.1)		—	
Charlson score, mean (SD)			2.7 (3.5)		1.2 (2.3)		—
≥1 ambulatory visit, n (%)			39,870 (99.3)		547,283 (93.8)		—
≥1 inpatient admission, n (%)			12,438 (31)		43,695 (7.5)		—
≥1 emergency department–only visit, n (%)			9131 (22.7)		75,913 (13)		—
≥1 emergency department–to-inpatient visit, n (%)			5143 (12.8)		24,757 (4.2)		—

^a^UCLA: University of California, Los Angeles.

^b^The Los Angeles County data reflect census data from 2019, which only provides rates [23].

^c^Not available.

Finally, the IPH patients were more likely to be male, married, and well educated compared to the UCLA Health population (Table 1).

Although the UCON video was available in Spanish, most individuals of self-reported Hispanic descent elected to complete the consent process in English (39,294/40,144, 97.9%; Table 2).

**Table 2:** Consent outcomes by workflow.

Consent outcomes		Perioperative and admission workflows (n=22,519), n (%)	Lab workflow (n=14,235), n (%)	Other or unknown (n=3390), n (%)	*P* value	
Consented in English		21,959 (97.5)	14,004 (98.4)	3331 (98.3)	<.001	
Self-administered consent		21,672 (96.2)	13,989 (98.3)	3325 (98.1)	<.001	
**Consent status**						<.001
	Extra tube or saliva sample	10,640 (47.2)	4047 (28.4)	1361 (40.1)		
	Remnant only	5860 (26)	3459 (24.3)	947 (27.9)		
	Declined	6019 (26.7)	6729 (47.3)	1082 (31.9)		
**Contact status**						<.001
	Do not contact	11,991 (53.2)	8166 (57.4)	1439 (42.4)		
	Contact	9545 (42.4)	4716 (33.1)	1152 (34)		
	Unknown	983 (4.4)	1353 (9.5)	799 (23.6)		

### Differences in Consent Across Clinical Settings

We observed a marked difference in the willingness to opt in between patients presenting to different clinics (perioperative suites: 16,500/22,519, 73.3%; ambulatory setting: 2308/3390, 68.1%; Table 2) and patients in the laboratory medicine workflow (7506/14,235, 52.7%; *P*<.001). Patients in perioperative settings were more likely to share an extra tube (10,640/22,519, 47.2%) than patients in other clinic settings (*P*<.001). The number of patients who decided to opt in and provide a dedicated extra tube was highest in perioperative suites (10,640/22,519, 47.2%), second highest in ambulatory settings (1361/3390, 40.1%), and lowest in clinical labs (4047/14,235, 28.4%; *P*<.001). Further, the number of patients who were willing to be recontacted was highest in perioperative suites (9545/22,519, 42.4%), second highest in ambulatory settings (1152/3390, 34%), and lowest in clinical labs (4716/14,235, 33.1%).

### Multivariate Analysis of Consent Variables

The most substantial differences in consent rates were observed in the perioperative setting. Those who were consented by using the laboratory workflow were significantly less likely to opt in than those who were consented by using the perioperative workflow (OR 0.43, 95% CI 0.42-0.46; *P*<.001; Table 3). There were also significant but less dramatic differences in opt-in rates based on self-reported ancestry; African American (OR 0.53, 95% CI 0.49-0.58; *P*<.001), Asian (OR 0.72, 95% CI 0.68-0.77; *P*<.001), and multiple-ethnicity populations (OR 0.73, 95% CI 0.69-0.77; *P*<.001) were less likely to opt in compared to those who self-identified as White (Table 3). More frequent health care utilization was also a significant predictor—those with greater than 1 inpatient admission had an OR of 1.28 (95% CI 1.22-1.35) for providing consent (*P*<.001). However, this was not the case for those who were frequent visitors of the emergency department, since those with greater than 1 emergency department visit had a lower consent rate than the average (OR 0.86, 95% CI 0.82-0.91; *P*<.001).

**Table 3:** Stepwise logistic regression model of any consent versus declines.

Variables			Unadjusted models				Multivariable model		
			Odds ratio (95% CI)		*P* value		Odds ratio (95% CI)		*P* value
**Race (reference: White)**					<.001				<.001
	Asian	0.66 (0.61-0.70)		<.001		0.72 (0.68-0.77)		<.001	
	Black or African American	0.52 (0.48-0.57)		<.001		0.53 (0.49-0.58)		<.001	
	Native Hawaiian or other Pacific Islander	1.06 (0.71-1.57)		.78		1.07 (0.71-1.60)		.76	
	American Indian or Alaska Native	0.77 (0.52-1.12)		.17		0.72 (0.49-1.07)		.10	
	Multiple races, other, unknown, or refused	0.67 (0.64-0.71)		<.001		0.73 (0.69-0.77)		<.001	
**Marital status (reference: married, significant other, or partner)**					<.001				<.001
	Single	0.81 (0.78-0.85)		<.001		0.89 (0.85-0.93)		<.001	
	Divorced, separated, dissolved, or widowed	1.00 (0.94-1.07)		>.99		0.95 (0.88-1.02)		.12	
	Other or unknown	0.60 (0.50-0.72)		<.001		1.00 (0.83-1.22)		.98	
≥1 inpatient admission			1.41 (1.34-1.47)		<.001		1.28 (1.22-1.35)		<.001
≥1 emergency department–only visit			0.90 (0.86-0.94)		<.001		0.86 (0.82-0.91)		<.001
**Workflow (reference: perioperative and admission workflows)**					<.001				<.001
	Lab workflow	0.41 (0.39-0.43)		<.001		0.43 (0.42-0.46)		<.001	
	Other or unknown	0.78 (0.72-0.84)		<.001		0.83 (0.76-0.89)		<.001	
Consented in English			0.72 (0.62-0.84)		<.001		0.74 (0.63-0.87)		<.001

We also tracked the number of patients who reached out to the biobank with questions about their participation (n=13). The low number of patients may suggest that the UCON process was self-explanatory and did not require additional support to complete; however, this was not measured directly.

## Discussion

In this study, we demonstrate that an integrated, institutional population–based electronic video consent process for the use of remnant biomaterials and deidentified phenotype data in research is feasible and scalable in large populations. The use of an animated, electronic video consent process is novel and innovative; ours is one of the few large-scale implementation studies that was conducted across a large health system and provided an enterprise solution for precision health research. Other reported efforts have been small, local, and nonrepresentative [24]. Compared to in-person paper consent, electronic video consent requires fewer human resources and less physical space and can be translated to reach diverse populations. A recent review of electronic consenting suggested that this modality is well received by participants, especially if it is accessible, user-friendly, and engaging and is tailored to specific patient populations [25,26]. The literature also suggests that this modality improves patient-centered outcomes, such as satisfaction and understanding [27]. At UCLA Health, we have implemented the translation of the UCON video into 8 languages that are represented widely across Los Angeles County. Other large precision health initiatives, such as the NIH All of Us Research Program, use more traditional consenting approaches and are limited to English and Spanish. In this regard, the expansion of the UCON process to several clinics across UCLA Health demonstrates the scalability of the UCON process as a recruitment engine that can power a large precision health program.

One of the goals of our precision health efforts is to ensure the inclusion of diverse populations and improve the accuracy of genetic prediction for all patients, regardless of ancestry. The inclusion of approximately 38.1% (15,304/40,144) of non-European patients in our biobank was likely achieved because we are located within one of the most diverse counties in the country. We note however that approximating the total diversity of Los Angeles County is a more daunting task that will require further, more significant outreach efforts. Although underrepresented populations are thought to have a lower willingness to participate in biobank research [28,29], community-based participatory research strategies have been shown to be effective [30-32]. Studies have shown that variations in the willingness to consent are mediated by different levels of trust in the health care and medical research system [33-35]. Patients are unsure about their rights over their biobank data [8] and have concerns about secondary research use [8,9]. Educating patients on the importance of genetic diversity for precision health approaches and creating toolkits to explain why their participation in large-scale programs is necessary will be important to the field.

This study also has its limitations. First, the UCON video was deemed to be at the ninth-grade reading level instead of the targeted seventh-grade reading level due to the use of many scientific terms and monosyllabic words. However, its success may reflect the demographics of the West Los Angeles population, which tends to be more educated and affluent. Despite this, we were successful in recruiting a diverse population for our expansion across UCLA Health. Future work will include working with community partners at our affiliated community hospitals to adapt the UCON process to communities with lower health and linguistic literacy. Our data are also slightly skewed toward older patients, as they make up a large proportion of patients in our procedure units (ie, those in ambulatory, laboratory medicine, and perioperative settings). Although we identified some sociodemographic and health characteristics that predicted the willingness to opt into the ATLAS program, our multivariate models, while predictive, did not explain all of the variance. As such, it is likely that there are considerable unmeasured factors that influence an individual patient’s decision process. The differences in consent rates across different clinical settings may suggest that patients are more willing to participate in biobank research in clinics, where there are higher rates of touch interactions between health care providers and patients.

Another potential limitation is that there was no measure for religiosity (how deeply religious an individual may be), which, in large national samples, has been shown to drive the willingness to participate in biobank research [36,37]. Additionally, our data only generalize to adults and do not generalize to pediatric patients. These populations often require specifically tailored consent and assent processes. Previously reported data show that patients’ willingness to consent differs from their willingness to allow their child to participate in research [37]. However, adolescents have a high capacity for consent—similar to normal adults—and consider themselves capable of making voluntary choices [38]. Future work has been planned with pediatric patients within UCLA Health to shed light on this issue. Lastly, the consenting process has not been tested across multiple institutions but rather represents a single institutional study conducted at UCLA, which resides in the large metropolitan area of Los Angeles where approximately 79% of adults aged >25 years have a high school diploma [23]. Discussions are underway to expand the consenting process to other University of California sites where using a standard consent process is important for collaboration and data sharing (eg, when federal and state requirements change or when conducting international studies in which a high level of consent is required) [29,39-41].

In conclusion, the use of the UCON process is a scalable and highly efficient approach for population-based consenting and the recruitment of patients for precision health research. This study shows that the UCON process can be deployed to any number of devices and implemented at multiple medical locations, making it suitable for large-scale efforts with modest incremental costs. Implementation strategies using this approach need to balance obtaining a large sample of consenting participants with ensuring a representative sample. Moreover, since this consenting approach is largely self-administered, combining our consent process with community outreach and education may be essential to reaching underserved populations and those that may have strong health beliefs about participation in research activities. We believe that our consent video and process offer an approach that allows for the more robust inclusion of institutions that do not have the financial resources for using employees to obtain in-person consent. Given the current reality that many such institutions serve patients who are chronically ill, are of lower socioeconomic status, and are from underrepresented populations, our consent video and process offer the possibility for these groups to become better represented in precision medicine research.

## Abbreviations

CAB: community advisory board
CTSI: Clinical and Translational Science Institutes
EHR: electronic health record
EngageUC: Engaging University of California Stakeholders for Biorepository Research
IPH: Institute for Precision Health
NIH: National Institutes of Health
OR: odds ratio
UCLA: University of California, Los Angeles
UCON: universal video consent

## References

[1] Ginsburg GS, Phillips KA (2018). Precision medicine: From science to value. Health Aff (Millwood).

[2] Petrini C (2010). "Broad" consent, exceptions to consent and the question of using biological samples for research purposes different from the initial collection purpose. Soc Sci Med.

[3] Greely HT (1999). Breaking the stalemate: a prospective regulatory framework for unforseen research uses of human tissue samples and health information. Wake Forest Law Rev.

[4] Federal policy for the protection of human subjects ('Common Rule'). U.S. Department of Health and Human Services, Office for Human Research Protections.

[5] NIH genomic data sharing policy. National Institutes of Health.

[6] (2015). NIH policy supports broader sharing of genomic data, strengthens informed-consent rules: research participants must give consent for secondary sharing, even if data are de-identified. Am J Med Genet A.

[7] Hens K, Nys H, Cassiman JJ, Dierickx K (2010). The use of diagnostic collections of DNA for research: interviews at the eight Belgian centers for human genetics. Eur J Med Genet.

[8] De Vries RG, Tomlinson T, Kim HM, Krenz C, Haggerty D, Ryan KA, Kim SYH (2016). Understanding the public's reservations about broad consent and study-by-study consent for donations to a biobank: Results of a national survey. PLoS One.

[9] Vermeulen E, Schmidt MK, Aaronson NK, Kuenen M, van der Valk P, Sietses C, van den Tol P, van Leeuwen FE (2009). Opt-out plus, the patients' choice: preferences of cancer patients concerning information and consent regimen for future research with biological samples archived in the context of treatment. J Clin Pathol.

[10] Rosa C, Campbell ANC, Miele GM, Brunner M, Winstanley EL (2015). Using e-technologies in clinical trials. Contemp Clin Trials.

[11] Simon CM, Schartz HA, Rosenthal GE, Eisenstein EL, Klein DW (2018). Perspectives on electronic informed consent from patients underrepresented in research in the United States: A focus group study. J Empir Res Hum Res Ethics.

[12] Boutin NT, Mathieu K, Hoffnagle AG, Allen NL, Castro VM, Morash M, O'Rourke PP, Hohmann EL, Herring N, Bry L, Slaugenhaupt SA, Karlson EW, Weiss ST, Smoller JW (2016). Implementation of electronic consent at a biobank: An opportunity for precision medicine research. J Pers Med.

[13] Simon CM, Klein DW, Schartz HA (2016). Interactive multimedia consent for biobanking: a randomized trial. Genet Med.

[14] Carey DJ, Fetterolf SN, Davis FD, Faucett WA, Kirchner HL, Mirshahi U, Murray MF, Smelser DT, Gerhard GS, Ledbetter DH (2016). The Geisinger MyCode community health initiative: an electronic health record-linked biobank for precision medicine research. Genet Med.

[15] Weitzel KW, Alexander M, Bernhardt BA, Calman N, Carey DJ, Cavallari LH, Field JR, Hauser D, Junkins HA, Levin PA, Levy K, Madden EB, Manolio TA, Odgis J, Orlando LA, Pyeritz R, Wu RR, Shuldiner AR, Bottinger EP, Denny JC, Dexter PR, Flockhart DA, Horowitz CR, Johnson JA, Kimmel SE, Levy MA, Pollin TI, Ginsburg GS, IGNITE Network (2016). The IGNITE network: a model for genomic medicine implementation and research. BMC Med Genomics.

[16] Vanaken H (2016). eConsent study provides insights to shape industry adoption. Appl Clin Trials.

[17] Abernethy ER, Campbell GP, Hianik R, Thomson MC, Dixon MD, Switchenko JM, Pentz RD (2020). Videos improve patient understanding of misunderstood chemotherapy terms in a rural population. J Clin Oncol.

[18] Dry SM, Garrett SB, Koenig BA, Brown AF, Burgess MM, Hult JR, Longstaff H, Wilcox ES, Madrigal Contreras SK, Martinez A, Boyd EA, Dohan D (2017). Community recommendations on biobank governance: Results from a deliberative community engagement in California. PLoS One.

[19] Naeim A, Dry S, Elashoff D, Xie Z, Petruse A, Magyar C, Johansen L, Werre G, Lajonchere C, Wenger N (2021). Electronic video consent to power precision research: A pilot cohort study. JMIR Form Res.

[20] Universal consent: What is universal consent? Sharing Biological samples, precision medicine. UCLA Health.

[21] Software. Epic.

[22] Charlson ME, Pompei P, Ales KL, MacKenzie CR (1987). A new method of classifying prognostic comorbidity in longitudinal studies: development and validation. J Chronic Dis.

[23] (2019). U.S. Census Bureau QuickFacts: Los Angeles County, California. United States Census Bureau.

[24] McDonald JA, Vadaparampil S, Bowen D, Magwood G, Obeid JS, Jefferson M, Drake R, Gebregziabher M, Hughes Halbert C (2014). Intentions to donate to a biobank in a national sample of African Americans. Public Health Genomics.

[25] Skelton E, Drey N, Rutherford M, Ayers S, Malamateniou C (2020). Electronic consenting for conducting research remotely: A review of current practice and key recommendations for using e-consenting. Int J Med Inform.

[26] Robins D, Brody R, Jeong IC, Parvanova I, Liu J, Finkelstein J (2020). Towards a highly usable, mobile electronic platform for patient recruitment and consent management. Stud Health Technol Inform.

[27] Golembiewski EH, Mainous 3rd AG, Rahmanian KP, Brumback B, Rooks BJ, Krieger JL, Goodman KW, Moseley RE, Harle CA (2021). An electronic tool to support patient-centered broad consent: A multi-arm randomized clinical trial in family medicine. Ann Fam Med.

[28] Garrison NA, Sathe NA, Antommaria AHM, Holm IA, Sanderson SC, Smith ME, McPheeters ML, Clayton EW (2016). A systematic literature review of individuals' perspectives on broad consent and data sharing in the United States. Genet Med.

[29] Trehearne A (2016). Genetics, lifestyle and environment. UK Biobank is an open access resource following the lives of 500,000 participants to improve the health of future generations. Bundesgesundheitsblatt Gesundheitsforschung Gesundheitsschutz.

[30] Passmore SR, Casper E, Olgin JE, Maguire C, Marcus GM, Pletcher MJ, Thomas SB (2019). Setting and motivation in the decision to participate: An approach to the engagement of diverse samples in mobile research. Contemp Clin Trials Commun.

[31] Hagiwara N, Berry-Bobovski L, Francis C, Ramsey L, Chapman RA, Albrecht TL (2014). Unexpected findings in the exploration of African American underrepresentation in biospecimen collection and biobanks. J Cancer Educ.

[32] Kim P, Milliken EL (2019). Minority participation in biobanks: An essential key to progress. Methods Mol Biol.

[33] Shavers VL, Lynch CF, Burmeister LF (2000). Knowledge of the Tuskegee study and its impact on the willingness to participate in medical research studies. J Natl Med Assoc.

[34] Kasperbauer TJ, Schwartz PH (2019). Measuring understanding and respecting trust in biobank consent. Am J Bioeth.

[35] Richter G, Krawczak M, Lieb W, Wolff L, Schreiber S, Buyx A (2018). Broad consent for health care-embedded biobanking: understanding and reasons to donate in a large patient sample. Genet Med.

[36] Sanderson SC, Brothers KB, Mercaldo ND, Clayton EW, Antommaria AHM, Aufox SA, Brilliant MH, Campos D, Carrell DS, Connolly J, Conway P, Fullerton SM, Garrison NA, Horowitz CR, Jarvik GP, Kaufman D, Kitchner TE, Li R, Ludman EJ, McCarty CA, McCormick JB, McManus VD, Myers MF, Scrol A, Williams JL, Shrubsole MJ, Schildcrout JS, Smith ME, Holm IA (2017). Public attitudes toward consent and data sharing in biobank research: A large multi-site experimental survey in the US. Am J Hum Genet.

[37] Antommaria AHM, Brothers KB, Myers JA, Feygin YB, Aufox SA, Brilliant MH, Conway P, Fullerton SM, Garrison NA, Horowitz CR, Jarvik GP, Li R, Ludman EJ, McCarty CA, McCormick JB, Mercaldo ND, Myers MF, Sanderson SC, Shrubsole MJ, Schildcrout JS, Williams JL, Smith ME, Clayton EW, Holm IA (2018). Parents' attitudes toward consent and data sharing in biobanks: A multisite experimental survey. AJOB Empir Bioeth.

[38] McGregor KA, Ott MA (2019). Banking the future: Adolescent capacity to consent to biobank research. Ethics Hum Res.

[39] Kaye J, Moraia LB, Curren L, Bell J, Mitchell C, Soini S, Hoppe N, Øien M, Rial-Sebbag E (2016). Consent for biobanking: The legal frameworks of countries in the BioSHaRE-EU project. Biopreserv Biobank.

[40] Lipworth W, Kerridge I (2015). Consent to biobank research: Facing up to the challenge of globalization. Am J Bioeth.

[41] Warner TD, Weil CJ, Andry C, Degenholtz HB, Parker L, Carithers LJ, Feige M, Wendler D, Pentz RD (2018). Broad consent for research on biospecimens: The views of actual donors at four U.S. medical centers. J Empir Res Hum Res Ethics.

